# Amiloride Sensitizes Prostate Cancer Cells to the Reversible Tyrosine Kinase Inhibitor Lapatinib by Modulating ERBB3 Subcellular Localization

**DOI:** 10.21203/rs.3.rs-4844371/v1

**Published:** 2024-08-30

**Authors:** Maitreyee K Jathal, Maria M Mudryj, Marc Dall’Era, Paramita M Ghosh

**Affiliations:** University of California Davis; University of California Davis; University of California Davis; University of California Davis

**Keywords:** Amiloride, lapatinib, ErbB3, subcellular localization, heregulin-1β, androgen receptor, prostate cancer

## Abstract

Neoadjuvant therapy (NAT) has been studied in clinically localized prostate cancer (PCa) to improve the outcomes from radical prostatectomy (RP) by ‘debulking’ of high-risk PCa; however, using androgen deprivation at this point risks castration resistant PCa (CRPC) clonal proliferation with potentially profound side effects such as fatigue, loss of libido, hot flashes, loss of muscle mass, and weight gain. Our goal is to identify alternative NAT that reduce hormone sensitive PCa (HSPC) without affecting androgen receptor (AR) transcriptional activity. PCa is associated with increased expression and activation of the epidermal growth factor receptor (EGFR) family, including HER2 and ErbB3. Dimerization between these receptors is required for activation of downstream targets involved in tumor progression. The FDA-approved HER2 inhibitor lapatinib has been tested in PCa but was ineffective due to continued activation of ErbB3. We now demonstrate that this is due to ErbB3 being localized to the nucleus in HSPC and thus protected from lapatinib which affect membrane localized HER2/ErbB3 dimers. Here, we show that the well-established, well-tolerated diuretic amiloride hydrochloride dose dependently prevented ErbB3 nuclear localization via formation of plasma membrane localized HER2/ErbB3 dimers. This in turn allowed lapatinib inactivation of these dimers via inhibition of its target HER2, which dephosphorylated downstream survival and proliferation regulators AKT and ERK1/2. Amiloride combined with lapatinib significantly increased apoptosis but did not affect AR transcriptional activity. Thus, our data indicate that a combination of amiloride and lapatinib could target HSPC tumors without problems associated with androgen deprivation therapy in localized PCa.

## INTRODUCTION

Localized prostate cancer (PCa) is initially often treated with radical prostatectomy (RP) or radiation therapy (RT), with an overall success rate of up to 90% alone (1). High grade and more advanced tumors, however, may require multi-modal therapy as 25–33% of men treated initially with these therapies eventually experience biochemical recurrence (2). While positive surgical margins at the time of RP are a risk factor for disease recurrence, many cancers are likely also micro-metastatic at the time of detection (3). Neoadjuvant therapy (NAT) has been studied to debulk tumors and treat early systemic disease prior to RP (4). NAT is also known to reduce post-operative residual local disease and micrometasis (5). This eliminates the need for salvage therapy after RP such as radiation or long term hormonal therapy (6).

PCa is initially dependent on the androgen receptor (AR), a nuclear hormone transcription factor activated by binding to androgens such as testosterone (T) and dihydrotestosterone (DHT) (7). With the advent of safe and reversible forms of androgen deprivation therapy (ADT) with or without antiandrogens, neoadjuvant ADT (NADT) is of significant interest (8). However, following prolonged exposure, many patients develop resistance to ADT, resulting in castration resistant PCa (CRPC) (7), which demonstrates the limitations of AR-based PCa therapy. Similarly, NADT also runs the risk of androgen-independent clonal proliferation with prolonged treatment (9). In addition, there may be impairments in quality of life due to profound side effects such as fatigue, loss of libido, hot flashes, loss of muscle mass, and weight gain (10). To overcome these problems, other therapies are being studied in the neoadjuvant setting, including PARP inhibitors, while tyrosine kinase inhibitors have been tried in preclinical and early clinical studies (11).

We and others have demonstrated that PCa is often associated with an increase in the activation of receptor tyrosine kinases (RTK) of the epidermal growth factor receptor (EGFR) family, including EGFR, HER2/ErbB2 and HER3/ErbB3 (HER4/ErbB4 is rarely expressed in PCa (12)) (13, 14). Our initial results showed that dual inhibition of EGFR and HER2 suppressed ErbB3 and sensitized PCa tumors to ADT (15). Members of the EGFR family are activated by ligand binding. EGFR has a number of ligands – including epidermal growth factor (EGF), while ErbB3 is activated by heregulins 1 and 2 (HRG1, HRG2) (16). Following ligand binding, these receptors undergo configurational alterations that allow heterodimerization with other members of the family. HER2 is known to be an orphan receptor that is constitutively active and hence does not require ligand binding for heterodimerization (16). For complete activation, all receptors undergo autophosphorylation at various tyrosine residues that bind downstream targets (16).

Many clinical trials have been conducted to evaluate the effects of FDA approved tyrosine kinase inhibitors (TKI) targeting EGFR in PCa – including cetuximab (17, 18), gefitinib (19–24) and erlotinib (25, 26). While a few trials showed moderate results in a subpopulation of PCa patients (e.g. erlotinib had moderate single-agent activity in chemotherapy-naïve CRPC (26), while cetuximab had some activity in those overexpressing EGFR and showing consistent expression of the tumor suppressor PTEN (17)), the majority of these trials failed to demonstrate efficacy. Of TKIs targeting HER2, pertuzumab was somewhat effective (27), while trastuzumab was ineffective as a single agent (28, 29) or in combination with the chemotherapeutic agent docetaxel (30, 31). In contrast, the dual EGFR/HER2 inhibitor lapatinib showed single agent activity in a small subset of patients (32). The advantage of the FDA approved lapatinib is its low toxicity and high tolerability (33). We previously showed that the pan-ErbB inhibitor dacomitinib was superior to lapatinib in preventing PCa progression (34); however, dacomitinib has greater side effects; hence, we investigated whether lapatinib efficacy could be improved with another low toxic drug.

We recently showed that ErbB3 is localized to the membrane/cytoplasm in benign prostate but shows nuclear translocation in malignant prostate (35). ADT increased ErbB3 cytoplasmic localization, whereas ErbB3 binding to its ligand heregulin-1β (HRG) induced ErbB3 nuclear localization (35). PCa-specific nuclear expression of ErbB3 has long been recognized (36). While an 80kDa nuclear variant of ErbB3 has been identified (37), full-length 185 kDa ErbB3 also translocates to the nucleus in PCa (38). Nuclear expression of the EGFR family is indicative of tumor development and progression in numerous tumor types (39, 40). We and others showed that increased nuclear localization of ErbB3 is associated with PCa progression (35, 41, 42).

Both EGFR and ErbB3 have been shown to undergo nuclear translocation through endocytosis (43–45). It is thought that internalization of ErbB3 initiates its entry into the nucleus where it interacts with the transcription complex and plays a role in transcriptional regulation, enabling PCa progression (44, 45). The macropinocytosis inhibitor amiloride hydrochloride has been shown to block ErbB3 nuclear translocation (45). Amiloride is used as a diuretic to treat hypertension (46–49). The plasma membrane localized sodium-hydrogen exchanger protein 1 (NHE1) (50), that plays a central role in intracellular pH and cell volume homeostasis, is a direct target of the drug (51). NHE1 activity is required to promote actin polymerization during macropinocytosis, explaining amiloride’s ability to antagonize this process (52). Amiloride can also prevent hypokalemia by inhibiting the epithelial sodium channel (ENaC) (53), and is therefore used as a potassium-sparing diuretic (54).

In this paper, we show that amiloride elicits a dose-dependent reduction in cell viability in hormone-sensitive PCa (HSPC). This is accompanied by a strong decrease in ErbB3 in the nuclear fraction and its accumulation in the cytoplasmic/membrane fraction in HSPC cells. Amiloride did not appear to have any appreciable effects on EGFR and HER2 localization. The efficacy of amiloride in decreasing cell viability was also enhanced by the simultaneous silencing of HER2 which dimerizes with ErbB3. Amiloride increased the efficacy of the reversible HER2 inhibitor lapatinib by increasing apoptosis in HSPC and CRPC cell lines, in agreement with previous reports in pancreatic cancer (55), and leukemia (56). Taken together, our data suggest that amiloride enhances lapatinib activity by limiting ErbB3 to the plasma membrane and/or cytoplasm and enabling HER2/ErbB3 dimerization, which allows lapatinib to inhibit the dimer and prevent downstream activation of Akt and ERK. Thus, this combination of lapatinib and amiloride will be considered a means of drug ‘re-purposing’, that is effective in HSPC, and hence may in the future be used in NAT.

## MATERIALS AND METHODS

### Cell culture and materials

Human prostatic carcinoma epithelial cell lines LNCaP, PC-346C and CWR22-Rv1 (ATCC, Manassas, VA) and C4–2 (MD Anderson, Houston, TX), were cultured in RPMI 1640 medium with 10% fetal bovine serum (Gemini Biologicals, West Sacramento, CA) and 1% antibiotic-antimycotic solutions (Gibco/Thermo Fisher Scientific, Waltham, MA). Lapatinib was purchased from Selleck Chemicals (Houston, TX). Amiloride hydrochloride was purchased from Amresco (VWR International, Radnor, PA). Heregulin-1β (HRG) was purchased from PeproTech (Rocky Hill, NJ). Rabbit monoclonal antibodies for EGFR (CS-2232), HER2 (CS-2165), ErbB3 (CS-12708) and Lamin A/C (CS-2032) were from Cell Signaling Technology (Beverly, MA). Mouse monoclonal antibody towards N-terminal ErbB3, OP-119 was purchased from Calbiochem/Millipore (San Diego, CA). ΜltraCruz Hard-set Mounting Medium was purchased from Santa Cruz BioTech (Dallas, TX).

### Subcellular fractionation

Cells were lysed for 15m at room temperature in 500–900μl of cytoplasmic lysis buffer A (10mM HEPES pH 7.9, 10mM KCl, 0.1mM EDTA, 0.4% IGEPAL) with standard protease and phosphatase inhibitors. The resulting suspension was centrifuged at 16000g for 5m at 4C and the supernatant was transferred to a clean 1.5ml tube and stored at −20C until further use. The pellet was washed thrice with 200–500μl 1X phosphate-buffered saline (Gibco, Thermo Scientific, Waltham, MA) (5m, 16000g, 4C) and reconstituted in ~ 150–300μl of 1X sodiμM dodecyl sμlphate (SDS) Sample Buffer (10g SDS, 4mls 100% glycerol, 40mls 1M Tris-HCl pH 8.8, made upto 100mls with doubly distilled water). The pellet was heated at 90C until it had completely dissolved, cooled to room temperature and stored at −20C until further use.

Immunofluorescence: LNCaP, C4–2, PC-346C or 22Rv1 cells were seeded at 10,000 cells per coverslip and were incubated for 24hrs in FBS medium in a 37°C CO_2_ incubator. Cells were treated with vehicle or drug for 72h, rinsed with PBST (Phosphate Buffered Saline with 0.05% Tween-20) and fixed with ice-cold methanol for 10 min on ice. They were washed three times with PBST and then blocked with 5% BSA for 1h at room temperature. Primary antibody was diluted 1:100 in 1% BSA and applied to the cells and incubated at 4°C overnight in a Humidity chamber. Cells were washed three times with PBST and the rhodamine or FITC-conjugated anti-rabbit or anti-mouse secondary antibodies (Life Technologies, Carlsbad, CA) were diluted 1:500 in PBST and incubated for 1 hr at room temperature in the dark. After washing thrice with cold PBST, coverslips were inverted and mounted onto uncharged glass slides with UltraCruz Hardset Mounting Medium plus DAPI (SantaCruz BioTech, Dallas, TX).

### 3-[4,5-Dimethylthiazol-2yl]-2,5-diphenyl-tetrazolium bromide assay

Cells were cultured in 24-well plates and treated as indicated. Following treatment, each well was incubated with 25 μl of 5 mg/ml 3-[4,5-dimethylthiazol-2yl]-2,5-diphenyl-tetrazoliuM bromide (MTT; Sigma–Aldrich, St. Louis, MO) for 1 h in a 5% CO2 incubator at 37 °C, which converted the reactants to formazan in actively dividing cells. Proliferation rates were estimated by colorimetric assay reading formazan intensity in a plate reader at 562 nm. Raw data are provided in Supplementary Fig. 7.

#### Western blotting:

Proteins were quantitated by BCA assay (Pierce, Rockford, IL, USA) and fractionated on 29:1 acrylamide-bis SDS–PAGE. Electrophoresis was performed at 150 V for 2 h using mini vertical electrophoresis cells (Mini-PROTEAN 3 Electrophoresis Cell, Bio-Rad). The gels were electroblotted for 2 h at 200 mA using Mini Trans-Blot Electrophoretic Transfer Cell (Bio-Rad) onto 0.2 μM polyvinylidene difluoride membrane (Osmonics, Westborough, MA, USA). The blots were stained overnight with primary antibodies at 4 °C and detected by enhanced chemiluminescence (Thermo Fisher, Waltham, MA) following incubation with a peroxidase-labeled secondary antibody (donkey anti-mouse IgG or goat anti-rabbit IgG, Fc specific, Jackson ImmunoResearch, West Grove, PA, USA).

### Invasion assay

LNCaP, C4–2 and CWR22-Rv1 cells were subjected to an invasion assay after being treated for 72h with the concentrations and combination of drugs as described on the y-axis of each graph. Falcon 8μm Transwell inserts were coated with 2μg/ml Matrigel (BD Biosciences) and cells allowed to invade through the basement membrane layer for 48h. Representative images (10X) are shown for the underside of each transwell insert after cells were fixed and stained with 0.5% v/v Crystal Violet in 100% methanol. Raw OD 595 readings were obtained after dissolving the intracellular Crystal Violet stain in 0.1% v/v acetic acid and colorimetric analysis at 595 nm.

Flow cytometry, plasmids, siRNA and transections were performed as described in detail previously by us (57).

## RESULTS

### Cytoplasmic/membranous retention of ErbB3 by amiloride in PCa cells correlates with its cytotoxic effects:

**Figure 1:**
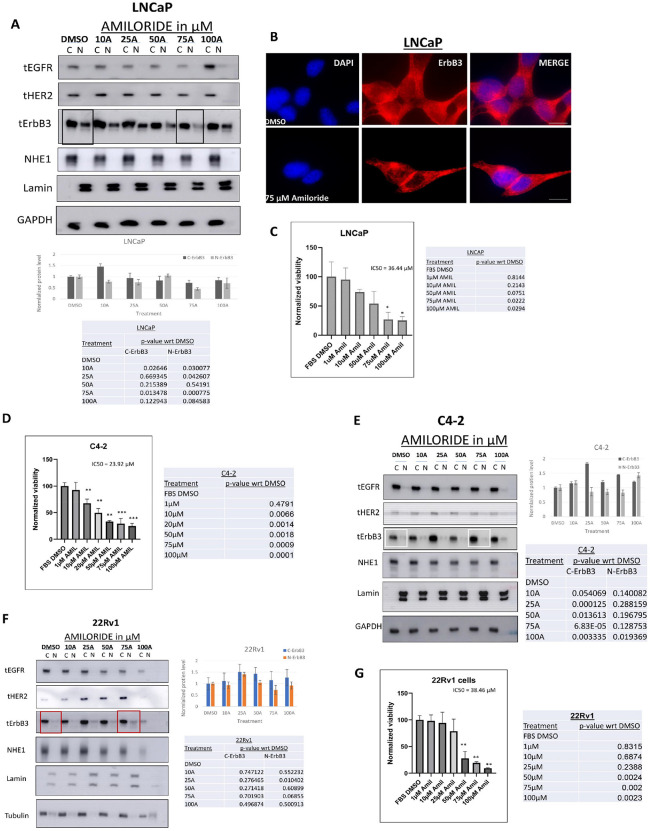
LNCaP cells are sensitive to amiloride and accumulate cytoplasmic ErbB3 upon amiloride treatment. **(A)**Hormone-sensitive LNCaP cells were treated with varying concentrations of amiloride for 72h before being lysed, fractionated and analyzed by immunoblot. **(B)**Immunofluorescence microscopy in LNCaP cells treated with DMSO or 75 μM amiloride for 72 hours (scale bars = 30μm). Note that vehicle treated LNCaP cells expressed nuclear ErbB3 (red) whereas amiloride-treated cells had significantly decreased ErbB3 expression in the nucleus (hollowed out). Location of nuclei are identified by blue DAPI staining. Plasma membrane localization of ErbB3 at cell-cell junction was also noted in amiloride-treated but not in vehicle treated cells. **(C)** Cells were subjected to viability assays using the stated concentrations of amiloride. Tables shows p-values with respect to DMSO. **(D,E)** Hormone-insensitive C4–2 or **(F,G)** the unrelated cell line 22Rv1 cells were also treated with varying concentrations of amiloride for 72h before being subjected to viability assays or lysed and fractionated as previously described. For all viability assays, results were obtained from triplicate experiments. Error bars represent standard deviation. Tables show p-values with respect to DMSO for each tested cell line. All densitometry was performed using ImageJ. C= cytoplasmic and N= nuclear.

We compared the effects of increasing concentrations of amiloride for 72h in hormone-sensitive LNCaP cells and their castration-resistant derivative C4–2 cells, as well as in an unrelated hormone-insensitive cell line CWR22Rv1 (denoted henceforth as 22Rv1). We have previously shown that all three cell lines express abundant ErbB3, HER2 and EGFR protein and mRNA (but not ErbB4) (34, 35). In LNCaP cells, baseline EGFR and HER2 were mostly cytoplasmic (Fig. 1A). ErbB3, in contrast, displayed both cytoplasmic and nuclear localization at baseline; however, at 75μM amiloride it was significantly cytoplasmic (p = 0.013) and only faintly nuclear (p = 0.00077) (Fig. 1A). This result was verified using immunofluorescence microscopy which showed nuclear ErbB3 in vehicle-treated cells but not in amiloride-treated cells as well as accumulation of ErbB3 at cell-cell junctions in amiloride treated cells (Fig. 1B). In parallel, amiloride also caused a dose dependent inhibition in cell growth, as indicated by MTT assay, with an IC_50_ = 36.44 μM (Fig. 1C), which is in the range previously reported as the optimal dose for amiloride (58) and corresponds to 50% of the dose at which amiloride eliminates nuclear ErbB3. Significant suppression of cell growth was observed at 75 μM (p = 0.0222), the dose at which ErbB3 translocated to the cytoplasm from the nucleus, suggesting correlation between loss of cell viability vs loss of nuclear ErbB3 localization.

### Differential activation of the EGFR family members and their downstream targets in HSPC and CRPC cells with high concentrations of amiloride:

**Figure 2:**
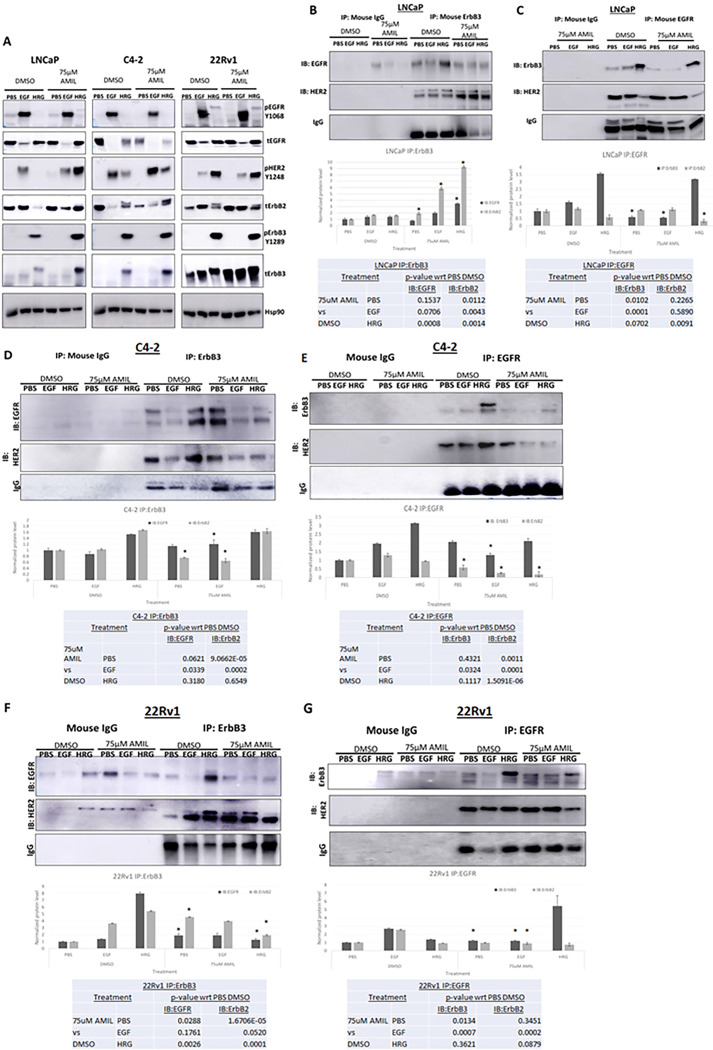
Differential activation and dimerization of ErbB family members and their downstream targets in HSPC and CRPC cells with high concentrations of amiloride **(A)** HSPC (LNCaP) and CRPC (C4–2, 22Rv1) cells were treated for 72h with 75μM amiloride dissolved in 100% sterile DMSO and stimulated with PBS, EGF or HRG for 15 minutes prior to collection to observe activation of ErbB family members and their downstream targets. Cells were lysed in denaturing lysis buffer before being analysed by immunoblotting. 25μg of protein were loaded per lane. Hsp90 was used as a loading control. **(B,C)** LNCaP cells (HSPC) were treated with 75μM amiloride or 100% DMSO (0.1% v/v) for 72h and stimulated with PBS, EGF or HRG for 15 minutes to activate ErbB family dimers just prior to collection. 400ug of whole cell lysate were used in each pulldown lane. Mouse IgG antibody was used as an isotype control. Amiloride increases ErbB3-HER2 dimers and stabilizes ErbB3-EGFR dimers. Densitometric analyses was performed with Image J for each cell lines tested. Tables show p-values with respect to DMSO for each tested cell line.

We next investigated the effects of amiloride on the phosphorylation status of the EGFR family and their prominent downstream targets, considered a measure of activation of these proteins. We previously showed that HRG1 stimulation of ErbB3 enables its translocation to the nucleus (35). Hence, we probed the appropriate phosphorylation sites that corresponded to activated ErbB receptors and their downstream targets in the presence or absence of 75μM amiloride. EGFR activation was determined by its phosphorylation at Y1068, a Grb2 binding site. EGF, but not HRG1, induced EGFR phosphorylation, and this effect was not altered by amiloride in any of the cell lines investigated (Fig. 2A). HER2 phosphorylation at Y1248 was, however, stimulated by both EGF and HRG1 in the CRPC lines, whereas it was mostly stimulated by EGF but not HRG1, in untreated LNCaP cells (Fig. 2A **left panel**). In the presence of 75 μM amiloride, however, HRG1 also stimulated HER2 phosphorylation at Y1248 in LNCaP cells. HRG1 but not EGF stimulated ErbB3 phosphorylation at Y1289, both in the presence and absence of amiloride. Similar to HER2 activation, ERK and Akt phosphorylation was induced by HRG1 only in the presence of amiloride in LNCaP cells, whereas in the C4–2 cells, amiloride induced ERK but not Akt phosphorylation. In 22Rv1 cells, amiloride did not induce such changes (**Supp.** Figure 3A). Transcript levels of any of the ErbB family members were largely unchanged across cell lines, except for EGFR mRNA in 22Rv1 cells which increased with 75μM amiloride (**Supp.** Figure 3B), but this is not reflected in the phosphorylation status of EGFR in 22Rv1 cells treated with 75 μM amiloride (Fig. 2A **right panel**). Thus, amiloride induced HER2 and ErbB3 phosphorylation by HRG1 selectively in LNCaP cells while this effect was significantly muted in CRPC cells. Together with the fact that ErbB3 is nuclear only in LNCaP cells at baseline, we conclude that nuclear ErbB3 precludes the activation of HER2 by HRG1 in these cells.

### Amiloride reduces HRG1-induced EGFR/ErbB3-heterodimers:

#### Effect of EGFR family receptor tyrosine kinases on ameliorating C4–2 cell viability is enhanced by amiloride treatment:

**Figure 3:**
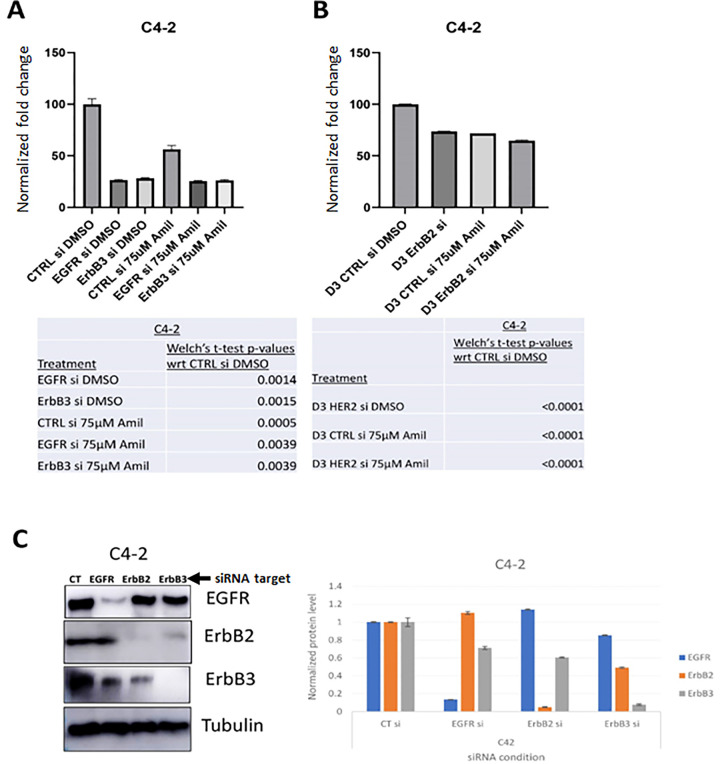
Amiloride efficacy is enhanced by EGFR knockdown in HSPC cells and by HER2 knockdown in CRPC cells. **(A)** CRPC C4–2 cells were transfected with control (CT) or EGFR or ErbB3 siRNA or **(B)** HER2 siRNA and treated with or without 75μM amiloride before being analysed for changes in viability with the MTT assay. Error bars represent standard deviation. Experiments were performed in triplicate. **(C)** Whole cell immunoblot for siRNA efficacy. 20μg of protein were loaded per lane. Tubulin was used as a loading control. Densitometry was performed using ImageJ. Error bars represent standard error of the mean.

Given the expression and stability of EGFR- and ErbB3-containing dimers and their activation of downstream signaling, we hypothesized that silencing either of these receptors would decrease cell viability. We used EGFR-, HER2- or ErbB3-specific silencing RNA (siRNA) sequences at a concentration of 10 pM per treatment condition to determine whether EGFR family receptors were involved in decreasing cell viability in response to amiloride treatment. The efficacy and specificity of EGFR family silencing has been previously assessed by us (57). Given the sensitivity of C4–2 cells to amiloride, these cells were used to test the effect of EGFR family on mediation of the effects of this drug. C4–2 cells showed significant decreases in viability with EGFR and ErbB3 siRNAs individually (approximately 75% decrease in viability using each siRNA) (p = 0.0015) (Fig. 3A). As before, amiloride significantly inhibited C4–2 cell growth, but knockdown of EGFR and ErbB3 in the amiloride treated cells had no further effect (Fig. 3A). However, knockdown of HER2 in C4–2 cells reduced viability by about 30% (p < 0.0001), whereas in amiloride treated cells, the same knockdown reduced viability by an additional 10% (p = 0.0041) (Fig. 3B). The efficacy of the siRNAs to EGFR, HER2, ErbB3 in C4–2 cells is shown in Fig. 3C. Thus, treatment with amiloride increased sensitivity to HER2 knockdown.

### Amiloride enhances the sensitivity of HSPC cells to lapatinib:

Amiloride directly inhibits growth factor receptor tyrosine kinase activity (59), is known to possess anti-cancer activity (51) and has been shown to enhance the efficacy of TKIs (55). We have previously shown that physiological concentrations of the FDA-approved reversible HER2 kinase inhibitor lapatinib were ineffective in inhibiting the growth of PCa cells (34). Since single-agent amiloride repressed viability and affected phosphorylation and dimerization levels of the EGFR family, we hypothesized that the addition of amiloride to lapatinib would enhance efficacy of the latter, at physiological doses of both drugs. This was necessary since hypokalemia (low potassium levels) is a common side effect of lapatinib (60), caused by excessive phosphorylation of hERG potassium channels (61), which may result in cardiac toxicity (62). while hyperkalemia is a side effect of amiloride (53, 54), and hence can counter this effect.

**Figure 4:**
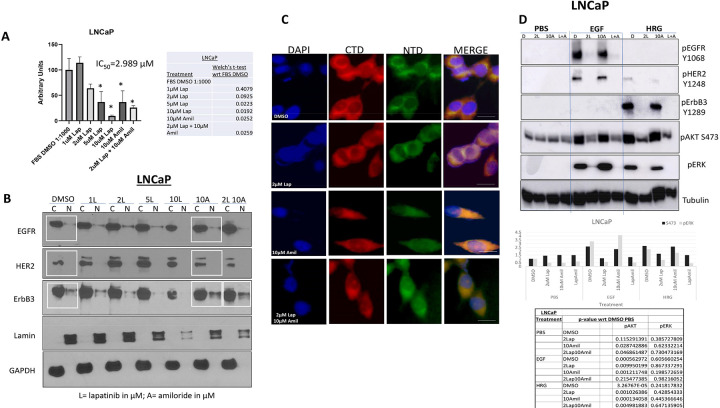
Amiloride enhances the sensitivity of HSPC cells to low concentrations of lapatinib **(A)** LNCaP cells were treated with 1–10μM lapatinib and assayed for viability. Lapatinib and amiloride were both dissolved in 100% DMSO. In a head-to-head comparison of lapatinib and amiloride, the combination was additive when cell viability was assayed with the MTT reagent. Experiments were done in triplicate. Error bars represent standard deviation. Table shows p-values with respect to FBS DMSO. **(B)** Cells were treated with varying concentrations of lapatinib, 10μM amiloride or a combination of the two for 72h before being collected and lysed into cytoplasmic and nuclear fractions as described in earlier figure legends. Co-administration of lapatinib and amiloride increases the accumulation of ErbB3 in the cytoplasmic fraction compared to 2μM lapatinib alone. **(C)** Cells were treated with 2μM lapatinib, 10μM amiloride or a combination of the two for 72h before being collected, fixed and processed for indirect immunofluorescent microscopy using immunofluorescent-specific antibodies to the C- and N- termini of ErbB3 (‘CTD’ and ‘NTD’ respectively) as previously described. Scale bars = 7.5 μm. **(D)** LNCaP cells were treated for 72h with lapatinib, amiloride or the combination or 100% sterile DMSO and stimulated with PBS, EGF or HRG for 15 minutes prior to collection to observe activation of ErbB family members and their downstream targets. Cells were lysed in denaturing lysis buffer before being analysed by immunoblotting. 25μg of protein were loaded per lane. Tubulin was used as a loading control.

We investigated the viability of LNCaP cells treated with increasing concentrations of lapatinib, a low dose of amiloride (10μM) or a combination of 2μM lapatinib and 10μM amiloride (‘2μM Lap + 10μM Amil’). The viability of LNCaP cells decreased only 36% with 2μM lapatinib (p = 0.0925) compared to 90.5% at the highest concentration of lapatinib tested (10μM, p = 0.0192). 10μM amiloride individually produced a reduction of 63.2% (p = 0.0252). When the two were combined however the resulting decrease was 74.2% (p = 0.0259) which was comparable to 10 μM lapatinib alone (p = 0.0091) (Fig. 4A). When the expression and localization of total protein levels of EGFR family members were analyzed, little change was seen in either parameter for EGFR or HER2 with either lapatinib or amiloride (Fig. 4B). In contrast, ErbB3 nuclear localization decreased in a dose-dependent manner from 0–10μM lapatinib (Fig. 4B), while ErbB3 remained nuclear with 10 μM amiloride with or without 2 μM lapatinib (Fig. 4B **and boxes within**). Correspondingly, immunofluorescent analysis of ErbB3 localization using an anti-C-terminal (CTD) and an anti-N-terminal (NTD) ErbB3 antibody depicted a strongly cytoplasmic and weak nuclear localization of ErbB3 structure under control conditions (Fig. 4C). 2 μM lapatinib did not disturb this pattern; however, with 10μM amiloride cells appeared elongated (Fig. 4C). At these concentrations, immunofluorescent imaging showed that with 2μM lapatinib treatment in the presence of amiloride, ErbB3 localization was cleared from the nucleoplasm but remained nucleolar. An immunoblot analysis of the signaling cascades under all the treatment conditions revealed, in agreement with Fig. 2A, that EGFR underwent phosphorylation (or activation) with EGF treatment, ErbB3 was phosphorylated with HRG1 and HER2 with both (but more strongly with EGF) under control conditions (Fig. 4D). With 10 μM amiloride, unlike 75 μM, there was no increase in HER2 phosphorylation, but there was no decrease in EGF-induced EGFR and HER2 phosphorylation nor a reduction in HRG1-induced ErbB3 phosphorylation (Fig. 4D). Significantly, lapatinib treatment, with or without amiloride, completely abrogated EGFR, HER2 and ErbB3 phosphorylation in LNCaP cells. This was accompanied by significant decreases in Akt phosphorylation and complete elimination of ERK phosphorylation as well (Fig. 4D).

**Figure 5:**
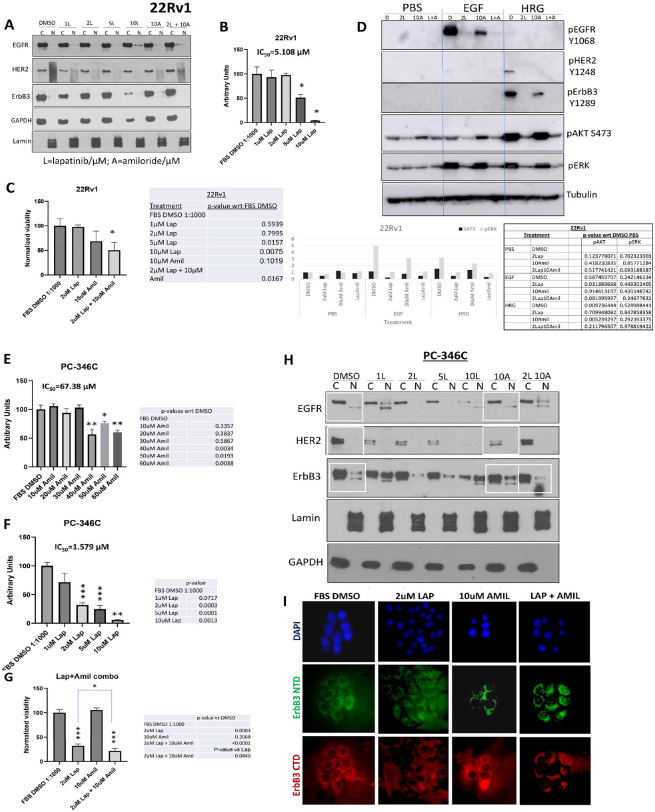
Amiloride enhances the sensitivity of HSPC and CRPC cell lines to low concentrations of lapatinib. **(A)** 22Rv1 cells were treated with varying concentrations of lapatinib, 10μM amiloride or a combination of the two for 72h before being collected and lysed into cytoplasmic and nuclear fractions as described in earlier figure legends. Co-administration of lapatinib and amiloride does not increase the accumulation of ErbB3 in the cytoplasmic fraction. **(B)** 22Rv1 cells were treated with 1–10μM lapatinib and assayed for viability. Lapatinib was dissolved in 100% DMSO. Experiments were done in triplicate. Error bars represent standard deviation. **(C)** In a head-to-head comparison of lapatinib and amiloride, the combination was additive when cell viability was assayed with the MTT reagent. Experiments were done in triplicate. Error bars represent standard deviation. Coloured dotted line estimates 50% viability. Table shows p-values with respect to FBS DMSO. **(D)** 22Rv1 (CRPC) cells were treated for 72h with lapatinib, amiloride or the combination or 100% sterile DMSO and stimulated with PBS, EGF or HRG for 15 minutes prior to collection to observe activation of ErbB family members and their downstream targets. Cells were lysed in denaturing lysis buffer before being analysed by immunoblotting. 25μg of protein were loaded per lane. Tubulin was used as a loading control. Tables show p-values with respect to PBS DMSO in each cell line tested. **(E)** PC-346C cells were treated with varying concentrations of amiloride and assayed for viability. Amiloride was dissolved in 100% DMSO. Experiments were done in triplicate. Error bars represent standard deviation. **(F)** PC-346C cells were treated with 1–10μM lapatinib and assayed for viability. Lapatinib was dissolved in 100% DMSO. Experiments were done in triplicate. Error bars represent standard deviation. **(G)** PC-346C cells were treated with lapatinib, amiloride or the combination, which is shown to be additive when cell viability was assayed with the MTT reagent. Experiments were done in triplicate. Error bars represent standard deviation. Table shows p-values with respect to FBS DMSO. **(H)** PC-346C cells were treated with varying concentrations of lapatinib, 10μM amiloride or a combination of the two for 72h before being collected and lysed into cytoplasmic and nuclear fractions as described in earlier figure legends.

In contrast to LNCaP cells, in 22Rv1 cells, which had very low baseline levels of nuclear ErbB3, this RTK remained cytoplasmic with lapatinib treatment, as well as with amiloride combinations, similar to EGFR and HER2 (Fig. 5A). These cells did however show a dose-dependent decrease in viability from 0–10μM lapatinib, with a 49% decrease with 5 μM lapatinib (p = 0.0157) and a 96% decrease at 10 μM lapatinib (p = 0.0075) with a resultant IC_50_ of 5.108 μM (Fig. 5B). However, the 10 μM intratumoral dose will not be physiologically relevant since achievement of that dose will put patients in conditions that will subject them to various adverse events prior to achieving that dose. To determine whether the more physiological dose of 2 μM can be enhanced by the addition of amiloride, we tested the combination of the two drugs in 22Rv1 cells (Fig. 5C). As before, 10 μM amiloride (that will not cause hyperkalemia) had by itself no significant effect on the viability of 22Rv1 cells (p > 0.05); however – the combination of 2 μM lapatinib and 10 μM amiloride reduced 22Rv1 viability by 49.9% (p = 0.0075). There was no significant effect on AR transcriptional activity at these drug concentrations (**Supplementary Fig. 5A**). As seen in LNCaP cells, lapatinib significantly suppressed the activation of the RTKs and their downstream targets, with or without the presence of amiloride (Fig. 5D) while amiloride but not lapatinib eliminated any nuclear ErbB3 that still may remain in these cells (**Supplementary Fig. 5B**).

Thus far, we have used three cell lines – that are sensitive to amiloride (IC_50_ in the range 20–40 μM), however, we then investigated the effect of the combination on a different cell line that is more resistant to amiloride. We have reported on PC-346C cells previously (13). These cells express wild type AR at very low levels and are considered to be hormone sensitive since they are inhibited by flutamide (63). We therefore tested the effect of the amiloride-lapatinib combination on PC-346C cells. This cell line was less sensitive to amiloride [IC_50_ = 67.38 μM (55.26 μM −108.3 μM)] (Fig. 5E) but was very sensitive to lapatinib [IC_50_ = 1.579 μM (1.203 μM −2.001 μM)] (Fig. 5F). While 10 μM amiloride had no effect on PC-346C cells, 2 μM lapatinib caused a 68% decrease in viability (p = 0.0003) (Fig. 5F). The combination of lapatinib and amiloride caused an additional 32.5% decrease in viability (p = 0.0440 compared to lapatinib alone) (Fig. 5G).

To determine whether the mechanism by which the combination works in a second hormone-sensitive PCa cell line PC-346C (64) is similar to that in LNCaP cells, we tested the effects of these treatments on EGFR, HER2 and ErbB3. Like the other lines, EGFR and HER2 was mostly cytoplasmic, and remained so, irrespective of the treatment. ErbB3 was partly nuclear, and the nuclear expression was enhanced by amiloride treatment, which explains its resistance to this drug (Fig. 5G). In contrast, lapatinib alone did not affect ErbB3 nuclear levels, but in the presence of amiloride, significantly reduced ErbB3 nuclear localization further, explaining the additive effect on cell viability (Fig. 5H). This is reinforced by immunofluorescent imaging showing that the combination of lapatinib and amiloride removes the levels of nuclear ErbB3 (Fig. 5I). Taken together, in hormone sensitive cells, the presence of nuclear ErbB3 induces resistance to reduction of viability, whereas in CRPC cells, where ErbB3 is not nuclear in the first place, this mechanism fails to have any significant effect.

### Amiloride and lapatinib synergize to induce apoptosis in HSPC cell lines:

**Figure 6:**
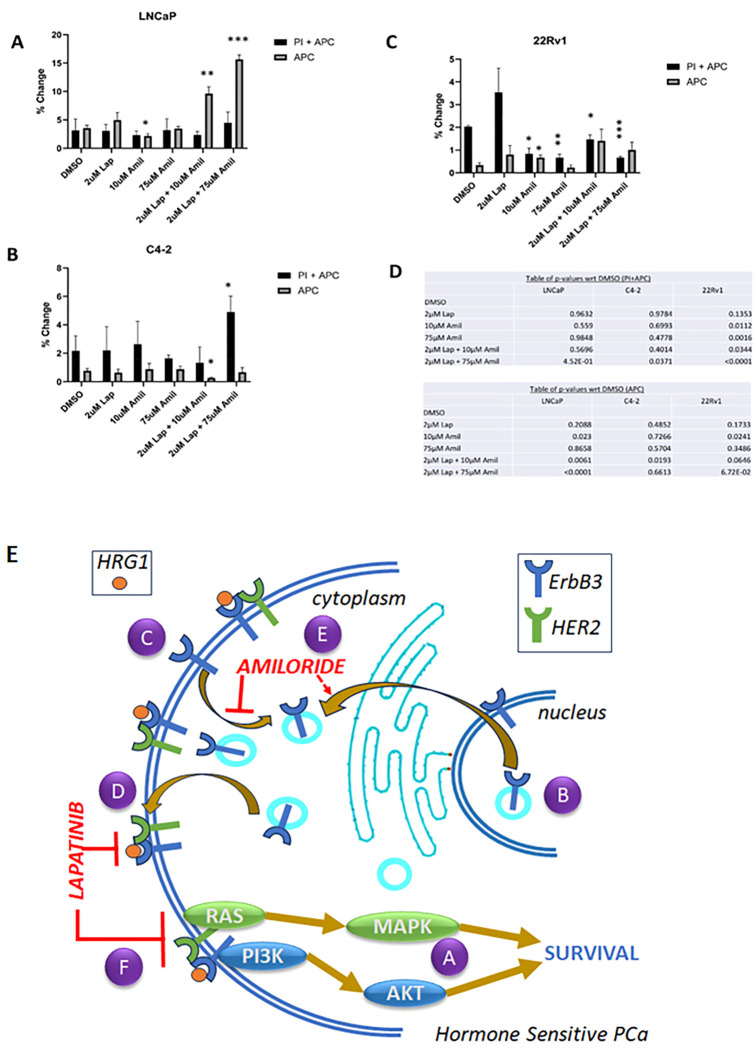
Amiloride and lapatinib synergize to increase apoptosis in HSPC and CRPC cell lines. **(A-C)** HSPC and CRPC cell lines were treated with 100% DMSO, lapatinib, amiloride or the combination (in μM) for 72h before being processed for cell death analysis using annexin V and propidium iodide staining. The percentage of cells undergoing early or late apoptosis with DMSO treatment was set to 100% and values for the various treatment conditions calculated accordingly. Experiments were performed in triplicate. **(D)** Tables show p-values with respect to DMSO in each cell line tested. **Schematic with proposed molecular mechanism of lapatinib-amiloride efficacy. (A)** EGFR, HER2 and ErbB3 exist at the cell membrane and signal via pathways such as ERK and AKT. **(B,C)** ErbB3 monomers cycle between the nucleus and cell membrane. (EGFR and HER2 behave similarly but have been omitted for clarity). **(D)** Lapatinib is a dual-kinase TKI (tyrosine kinase inhibitor) of HER2 and EGFR dimers but will also inhibit HER2 in HER2-ErbB3 dimers. Lapatinib is unlikely able to inhibit ErbB3 if it is in the nucleus and not at the cell surface. **(E)** Amiloride is a macropinocytosis inhibitor that prevents internalization of ErbB3 and retains it at the cell surface. As a result, nuclear ErbB3 decreases and cytoplasmic surface ErbB3 increases. **(F)** Amiloride-induced ErbB3 retention enables its dimerization with HER2, enabling the formation of ErbB3-HER2 dimers which are now inhibited by the addition of low concentrations of lapatinib.

The goal of cancer treatment is to ensure that all malignant cells are dead, not dormant. However, the changes in cell viability that we have conducted thus far could be due to an increase in apoptosis, or the onset of various mechanisms that may have led to cellular quiescence. Hence, we conducted cell death analyses to ascertain the mechanism causing the consistent decreases in viability seen in all 3 cell lines with the combination of low dose lapatinib and amiloride. Flow cytometry was employed using DNA-bound propidium iodide (PI) as a necrosis marker and cell surface expression of Annexin V using the Annexin V-Allophycocyanin (APC) conjugate as a marker of apoptosis. LNCaP cells showed no significant change in early apoptosis (‘APC’) or late apoptosis (apoptosis with necrosis, ‘PI + APC’) with 2μM lapatinib and a slight decrease in early apoptosis (−39.6%) with 10μM amiloride (p = 0.023) (Fig. 6A). The combination of 2μM Lap + 10μM amiloride produced a sharp increase in the percentage of cells undergoing early apoptosis (2.7-fold, p = 0.0061) and this was increased further when amiloride was used at 75μM (4.42-fold, p < 0.0001), although no change in the fraction of cells in late apoptosis was noted (Fig. 6A). In contrast, C4–2 cells exhibited an increase in necrotic cells only when 2 μM lapatinib + 75μM amiloride were used (2.26-fold, p = 0.0371) which may indicate toxicity, rather than programmed cell death, while a combination of 2 μM lapatinib + 10μM amiloride actually resulted in a 65% decrease in apoptosis (p = 0.0193) (Fig. 6B). Similar to C4–2 cells, 22Rv1 cells displayed no change in apoptosis when exposed to 2μM lapatinib either alone or in combination with 10 μM or 75 μM amiloride, and significantly reduced cell death with 2μM lapatinib in combination with both 10μM and 75μM amiloride (Fig. 6C). Representative raw readings for these data are provided in supplementary information (**Supp.** Figures 6–8). Thus, in LNCaP cells, the decrease in cell viability with the combination of 2 μM lapatinib + 75μM amiloride observed is likely due to an increase in apoptosis while that in C4–2 cells, any change in viability is likely caused by increase in toxicity, and no substantial effects of the combination was observed in 22RV1 cells. Taken together, this indicates that the combination of 2 μM lapatinib + 10μM amiloride was effective in inducing programmed cell death in HSPC LNCaP cells (higher doses may cause toxicity), but not in CRPC lines at any dose.

## DISCUSSION

Here we present a novel treatment strategy for inhibiting hormone sensitive PCa using amiloride to enhance the cytotoxicity of the FDA-approved reversible dual TKI lapatinib that in the future may be studied in a neoadjuvant setting for clinically localized, high-risk PCa. Lapatinib possesses several advantages as a therapeutic strategy for neoadjuvant therapy – it is well-established, relatively well tolerated (with mild diarrhea and rash being the most common toxicities) and easy to administer orally (33). Major pathways involved in its efficacy and resistance have been identified and investigated (65). Lapatinib is most effective in settings where members of the epidermal growth factor receptor (EGFR) family are overexpressed, its specific targets being HER2/ErbB2 and EGFR (66). Lapatinib is designed to inhibit the kinase domains of these receptors after they have dimerized, preventing receptor auto and transphosphorylation and subsequent downstream activation of proliferation and survival pathways, for example via ERK/MAPK and AKT respectively (67). Lapatinib was shown to bind to lipid membranes and insert into the lipid-water interface of the bilayer (68). Therefore, it is necessary for the kinase domains of EGFR and HER2 to be accessible to lapatinib and this is most likely to occur when they are situated in the cytoplasm or plasma membrane of tumor cells.

It has been well-documented that lapatinib is most effective on HER2/ErbB3 dimers. Lapatinib induces HER2/ErbB3 dimers (69), and increased HRG and activated ErbB3 strongly correlated with lapatinib sensitivity (70). In this paper, we demonstrate that while ErbB3 fluctuated between the plasma membrane, the cytosol and nucleus, HER2 was primarily located in the plasma membrane. This is reasonable since ErbB3 was previously shown to endocytose to the cytosol via clathrin-dependent mechanisms while it’s transportation to the nucleus required binding to importin β (43). In contrast, HER2 translocated to the nucleus in a manner dependent on HSP90 binding (71). Thus, for the HER2/ErbB3 dimer to form, it would follow that ErbB3 would need to be located on the plasma membrane of PCa cells. This poses a challenge for PCa, where ErbB2/HER2 is expressed on the plasma membrane but ErbB3/HER3 is significantly nuclear (35). Hence in order for HER2/ErbB3 dimers to readily form, potentially to increase lapatinib efficacy, ErbB3 needs to be coaxed to come to the plasma membrane and be retained there long enough to enable dimer formation and downstream action.

We demonstrate that amiloride causes a dose dependent translocation of ErbB3 from a nuclear to a cytoplasmic localization only in HSPC LNCaP cells, that coincides with a dose dependent loss of cell viability. Investigation of the phosphorylation status of these RTKs demonstrated that nuclear ErbB3 precludes the activation of HER2 by HRG1, which stimulated HER2 and ErbB3 phosphorylation predominantly in LNCaP. Co-immunoprecipitation and immunofluorescence studies suggested that amiloride realigned HER2 from EGFR to ErbB3 containing dimers in LNCaP cells. Knockdown of the RTK genes demonstrated that amiloride enhanced the cell killing properties associated with HER2 knockdown. Hence, we used the HER2 inhibitor lapatinib to enhance the loss of viability induced by amiloride. The combination enabled the use of the drugs at much lower doses to prevent adverse cellular toxicity. We demonstrated that in LNCaP cells, but not in CRPC cells, this loss of viability was caused by an increase in apoptosis. Taken together, these results indicate that amiloride induces apoptosis in HSPC cells by enabling ErbB3 accumulation in the cytoplasmic/membranous domain, where it can be inhibited by lapatinib.

High concentrations of amiloride dose-dependently decreased nuclear ErbB3 and increased cytoplasmic ErbB3 almost entirely in LNCaP (HSPC) cells. The CRPC lines C4–2 and 22Rv1 did not display this effect and some reasons are: (1) ErbB3 was predominantly cytoplasmic in these cells to begin with (2) ErbB3 was resistant to amiloride because a pathway other than macropinocytosis was involved in internalization or (3) an RTK other than ErbB3 was the target. Indeed, in 22Rv1 cells we observed a dose-dependent reduction in cytoplasmic EGFR protein but a compensatory increase in transcript levels. Significantly, The Human Protein Atlas indicates that ErbB3 is expressed in PCa at much higher levels than EGFR or HER2 (72). Therefore, it is likely that in these tumors, EGFR and HER2 reside primarily in the plasma membrane whereas ErbB3 shuttles back and forth between different cellular components as needed.

The EGFR family of RTKs signals primarily at the plasma membrane after ligand binding; hence, our next steps were to analyze the effects of amiloride on dimerization and phosphorylation (used as markers for activation) of this family as well as its downstream targets ERK and AKT. RTK phosphorylation was induced by treatment with specific ligands epidermal growth factor (EGF) or heregμlin-1β (HRG), which activate EGFR and ErbB3 respectively and influence their binding to HER2. In LNCaP cells, 75μM amiloride caused the retention and accumulation of activated, membrane-bound ErbB3, which dimerized with activated HER2 and signaled via both ERK and AKT. The amiloride-induced ErbB3/HER2 dimers increased, and were not disrupted in the presence of EGF or HRG. In C4–2 cells, 75μM amiloride + EGF decreased EGFR/HER2 dimers but not ERK phosphorylation. 75μM amiloride also prevented dimerization of HER2-ErbB3 and HER2-EGFR in these cells. Protein levels of phosphorylated EGFR family members and their downstream targets were unchanged in 22Rv1 cells. Levels of total protein for EGFR and HER2 were largely unaffected by 75μM amiloride in all cell lines, unlike total ErbB3 protein, which was significantly increased in all cell lines, including 22Rv1. This resulted in differential AKT and ERK signalling in LNCaP cells, ERK signalling in C4–2 cells (AKT phosphorylation was unaffected) and constitutive AKT and ERK signalling in 22Rv1 cells. From these results we learned that modulation of ErbB3 localization influenced dimer formation with consequences for downstream target activation in HSPC vs CRPC cell lines which might impact tumor cell proliferation (ERK signalling) and survival (AKT signalling).

If 75μM amiloride modulates the localization and activation of the ErbB family and consistently decreases viability, then we wondered whether silencing these RTKs would enhance its inhibitory effects. Using sequence-specific siRNA described by us previously (57), we observed that silencing ErbB2 (but not EGFR or ErbB3) enhanced the inhibitory effect of amiloride in C4–2 cells. Thus, we tested our hypothesis that the enhanced inhibitory effect of amiloride, when combined with ErbB2 knockdown, would also be evident if ErbB2 was inhibited with the use of the dual-kinase HER2/EGFR inhibitor lapatinib. The concentration of lapatinib (2μM) was used as previously described (57). In LNCaP and 22Rv1 cells, a combined low dose of amiloride and lapatinib significantly decreased cell viability over either drug alone. Protein levels of the EGFR family were largely membrane bound, except for ErbB3 in LNCaP cells, which remained nuclear under the conditions where viability was decreased the most (Lap + Amil). While indirect immunofluorescent microscopy in LNCaP cells could not sharply discriminate localization patterns between cytoplasmic and nuclear ErbB3, we noticed the presence of a ‘ring’-like structure with both N- and C-terminal ErbB3 antibodies and infer that it denotes full-length nuclear ErbB3, as was previously observed in our patient tissues (35).

The combination of Lap + Amil decreased cell viability by significantly increasing early apoptosis. We noted with interest that in LNCaP cells, apoptosis was observed when ErbB3 was entirely cytoplasmic and its nuclear expression was minimal. From this we reason that lapatinib increases cytoplasmic HER2 and amiloride inhibits macropinocytosis of ErbB3 causing its cytoplasmic accumulation and preventing its nuclear expression. ErbB3 thus confined to the plasma membrane dimerizes with HER2, enabling the formation of active ErbB3/HER2 dimers, whose kinase domains are now targeted by the TKI lapatinib, whose primary mechanism of action is inhibition of cytoplasmic RTK dimers. Having observed that ErbB3/HER2 is the most prominent dimer in amiloride-treated LNCaP cells, this is a plausible explanation for the significant increase in apoptosis induced by the Lap + Amil combination in this cell line. Thus, we conclude that the combination of amiloride followed by lapatinib would likely reduce tumor volume selectively in HSPC tumors, and therefore may be studied as neoadjuvant therapy to improve outcomes from RP.

There may even be some additional effects of the combination. Although lapatinib is usually well tolerated, it has been reported to have cardiotoxic effects in some due to hypokalemia caused by continuous diarrhea. Amiloride, being a potassium sparing diuretic, may prevent this effect by reinforcing hyperkalemia. Both are oral drugs that can be easily administered. Hence additional studies may be warranted to test the efficacy of the combination in a neoadjuvant setting.

## Data Availability

All available data has been presented in the manuscript or in the Supplementary Materials. If additional detail is requested, the authors may be contacted for further information. All materials generated for this paper, if available, can be shared with interested individuals against an MTA with the University of California, Davis.

## Abbreviations

PCa: prostate cancer
AR: androgen receptor
FBS: fetal bovine serum
CSS: charcoal stripped serum
HRG-1β: heregulin-one-beta
EGF: epidermal growth factor
DHT: dihydrotestosterone
HSPC: hormone sensitive prostate cancer
CRPC: castration resistant prostate cancer
PSA: prostate specific antigen
mRNA: messenger RNA
